# Modeling of H_2_S solubility in ionic liquids: comparison of white-box machine learning, deep learning and ensemble learning approaches

**DOI:** 10.1038/s41598-023-34193-w

**Published:** 2023-05-16

**Authors:** Seyed-Pezhman Mousavi, Reza Nakhaei-Kohani, Saeid Atashrouz, Fahimeh Hadavimoghaddam, Ali Abedi, Abdolhossein Hemmati-Sarapardeh, Ahmad Mohaddespour

**Affiliations:** 1 Department of Petroleum Engineering, Shahid Bahonar University of Kerman, Kerman, Iran; 2 Department of Chemical and Petroleum Engineering, Shiraz University, Shiraz, Iran; 3 Department of Chemical Engineering, Amirkabir University of Technology (Tehran Polytechnic), Tehran, Iran; 4 Key Laboratory of Continental Shale Hydrocarbon Accumulation and Efficient Development, Ministry of Education, Northeast Petroleum University, Daqing, 163318 China; 5 Ufa State Petroleum Technological University, Ufa, 450064 Russia; 6 College of Engineering and Technology, American University of the Middle East, Egaila, 54200 Kuwait; 7 State Key Laboratory of Petroleum Resources and Prospecting, China University of Petroleum (Beijing), Beijing, China; 8 Department of Chemical Engineering, McGill University, Montreal, QC H3A 0C5 Canada

**Keywords:** Chemical engineering, Computational methods

## Abstract

In the context of gas processing and carbon sequestration, an adequate understanding of the solubility of acid gases in ionic liquids (ILs) under various thermodynamic circumstances is crucial. A poisonous, combustible, and acidic gas that can cause environmental damage is hydrogen sulfide (H_2_S). ILs are good choices for appropriate solvents in gas separation procedures. In this work, a variety of machine learning techniques, such as white-box machine learning, deep learning, and ensemble learning, were established to determine the solubility of H_2_S in ILs. The white-box models are group method of data handling (GMDH) and genetic programming (GP), the deep learning approach is deep belief network (DBN) and extreme gradient boosting (XGBoost) was selected as an ensemble approach. The models were established utilizing an extensive database with 1516 data points on the H_2_S solubility in 37 ILs throughout an extensive pressure and temperature range. Seven input variables, including temperature (T), pressure (P), two critical variables such as temperature (T_c_) and pressure (P_c_), acentric factor (ω), boiling temperature (T_b_), and molecular weight (Mw), were used in these models; the output was the solubility of H_2_S. The findings show that the XGBoost model, with statistical parameters such as an average absolute percent relative error (AAPRE) of 1.14%, root mean square error (RMSE) of 0.002, standard deviation (SD) of 0.01, and a determination coefficient (R^2^) of 0.99, provides more precise calculations for H_2_S solubility in ILs. The sensitivity assessment demonstrated that temperature and pressure had the highest negative and highest positive affect on the H_2_S solubility in ILs, respectively. The Taylor diagram, cumulative frequency plot, cross-plot, and error bar all demonstrated the high effectiveness, accuracy, and reality of the XGBoost approach for predicting the H_2_S solubility in various ILs. The leverage analysis shows that the majority of the data points are experimentally reliable and just a small number of data points are found beyond the application domain of the XGBoost paradigm. Beyond these statistical results, some chemical structure effects were evaluated. First, it was shown that the lengthening of the cation alkyl chain enhances the H_2_S solubility in ILs. As another chemical structure effect, it was shown that higher fluorine content in anion leads to higher solubility in ILs. These phenomena were confirmed by experimental data and the model results. Connecting solubility data to the chemical structure of ILs, the results of this study can further assist to find appropriate ILs for specialized processes (based on the process conditions) as solvents for H_2_S.

## Introduction

Hydrogen sulfide (H_2_S) exists in abundance in various gas fields, including synthesis gas, natural gas, and refinery gas, as well as in crude oil hydrodesulfurization operations^1,2^. Hydrogen sulfide must be eliminated from industrial processes due to its high toxicity, flammable gas, and acidity. H_2_S sequestration from natural gas generated from gas reservoirs is required to conform with environmental legislation, safety requirements, and selling gas production standards^2–4^. The most widely suggested approaches utilized in gas refineries and other associated industries are amine-based remedial activities, which employ aqueous systems of various alkylamines to discrete H_2_S and CO_2_^2–7^. Absorption in alkanolamine-based solvents is now a commonly utilized technique for the elimination of acid ingredients. For this process, several industrially significant alkanolamines, namely diethanolamine (DEA), di-isopropanol-amine (DIPA), *N*-methyl-diethanolamine (MDEA), and mono-ethanolamine (MEA) are used. During the absorbent process, however, the alkanolamine solutions have certain drawbacks, including high economic expense, high energy utilization, and caustic by-product. As a result, to properly remove H_2_S, environmentally friendly and highly efficient absorbents are required^4,5^.

Ionic liquids (ILs) have recently earned much attentiveness as a suitable replacement for alkanolamine absorbents^8^. ILs are a novel family of solvents that have several prominent characteristics, such as non-inflammability, trivial vapor pressure, a vast liquid range, excellent temperature stability, good ion and electrical conductivity, electrochemical consistency, simplicity of recycling, high viscosity, and adjustable properties^9–12^. As a result, they have the potential to be highly useful in chemical processes, and petroleum engineering. Like salt molecules, ILs are formed of an anion (namely; tetrafluoroborate, chloride, and hexafluorophosphate anions) and a cation (like pyridinium and ammonium cations)^13^. Because of the small vapor pressure of ILs compared to that of other solvents, they are referred to as polar non-volatile solvents^14,15^. To develop, optimize, and regulate gas–liquid absorption processes, an exhaustive cognition of the gas solubility in ILs at different thermodynamic conditions is required^16,17^.

Knowing the gas solubility at various thermodynamic conditions is a key part of evaluating ILs for prospective utilization in gas sweetening systems^16,18^. Despite numerous recent papers that have been published on gas solubility in ILs, particularly CO_2_, real H_2_S/ILs solubility data is infrequent. This may be because of the time-consuming quiddity of laboratory measurements, the high toxicity of H_2_S, and the high cost of the procedures. As a result, establishing predictive techniques for evaluating the attributes of such systems under a variety of scenarios is highly recommended^19–24^.

The use of empirical correlations^25,26^, equations of state (EOSs)^27–33^, and molecular descriptors^34,35^ to investigate and model H_2_S solubility has already been discussed in the literature. These methods are usually restricted to a given system and composition, as well as a defined temperature and pressure range. Systems based on Artificial Intelligence (Al) and Machine Learning (ML) approaches can be appropriate and vigorous in calculating the H_2_S solubility in ILs since they can be constructed on data from an extensive range of materials under varied thermodynamic circumstances, particularly if a thorough database is utilized for model building^36–38^. Furthermore, AI and ML approaches have improved precision and generality when modeling a variety of phenomena in science and engineering domains such as environmental engineering^39^, petroleum engineering^40–45^, physical chemistry^46–48^, civil engineering^49^, earth science^49^, and chemical engineering^46,49–56^.

Many ILs have been synthesized during the last few decades using various types of cations and anions, and the solubility of various gases, such as O_2_, NH_3_, SO_2_, CO, N_2_, CO_2_, and, H_2_S in them has been studied at various temperatures and pressures^57^.

Based on 465 data points acquired from the published works, Ahmadi et al.^58^ presented a gene expression programming (GEP) system for the calculation of H_2_S solubility in ILs, where several thermodynamic properties, namely acentric factor (ω), critical pressure (P_c_), pressure (P), critical temperature (T_c_), and temperature (M_w_) were selected as inputs. The results reveal that Soave–Redlich–Kwong (SRK) and Peng–Robinson (PR) EOSs are less accurate than the designed GEP model, which has an average absolute percent relative error (AAPRE) value of 0.04%. Shafiei et al.^59^ also used their databank to analyze particle swarm optimization (PSO) trained systems and backpropagation (BP). According to the findings, the PSO-ANN method was more precise than those of the BP-ANN system with an AAPRE value of 4.58%. This databank was also used by Ahmadi et al.^60^ and the findings of the least-squares support vector machine coupled with a genetic algorithm (LSSVM-GA) approach resulted in an AAPRE value of 7.01%. Amedi et al.^61^ developed three ML approaches to forecast H_2_S solubility in different ILs with parameters such as the T_c_, M_w_, and P_c_ of pure ILs for 664 solubility data points: Radial basis function (RBF), Multilayer perceptron (MLP), and Adaptive neuro-fuzzy inference system (ANFIS). Statistical evaluations indicated that the ANFIS, MLP, and RBF approaches, respectively, had mean absolute errors of 0.021, 0.0089, and 0.042. Zhao et al.^62^ reported that an extreme learning machine (ELM) system established with a large dataset including 1282 data points could accurately predict H_2_S/ILs solubility with an AAPRE value of 4.12%. Baghban et al.^63^ proposed an LSSVM method to predict the H_2_S solubility based on the chemical structure of cation and anion, P, and T with a vast data bank including 1282 solubility data, where the model's AAPRE value was 2.74%. Hosseini et al.^17^ employed 1243 data points in 33 different ionic liquids to develop an MLP model for calculating H_2_S solubility. Some factors were considered in this work, including pressure, acentric coefficient, and critical temperature, and the findings revealed that the proposed system can be utilized to predict H_2_S solubility with an absolute error of 2.57%. With the equal inputs as the prior work, Menad et al.^18^ proposed a novel committee machine intelligence system with genetic programming (CMIS-GP) to compute the hydrogen sulfide solubility in 33 ILs (1243 experimental data). The results demonstrate that the CMIS-GP model has a higher accuracy than the other proposed models, with an AAPRE of approximately 2.37%. Mousavi et al.^53^ established a convolutional neural network (CNN) model to estimate H_2_S solubility in ILs using constituent substructures of ILs and operating parameters with a large dataset containing 1516 data for 37 ILs. The result demonstrated that the CNN system can determine H_2_S solubility better than other methods, with an AAPRE value of 2.92%. Zhang et al.^64^ suggested a least-squares boosting (LSBoost) model based on P_c_, T_c_, P, the acentric factor of the ILs, and the temperature of biphasic systems to determine the solubility of supercritical CO_2_ in 24 ILs. The findings demonstrated that the developed LSBoost paradigm had RMSE and MAE of 2.78 and 1.62%, respectively. Zhang et al.^65^ designed a Gaussian process regression (GPR) model to inquire about the relationship between temperature, nanoparticle volume concentration and specific heat capacity, and the specific heat capacities of nanofluids and base liquids. The results demonstrated a high estimation precision as reflected in the estimation mean absolute error (MAE) and root mean square error (RMSE) which were 0.05% and 0.06% of the average experimental ${C}_{p-nf}$, respectively. Mosavi et al.^66^ implemented two bagging models and two boosting models for groundwater potential estimation with 339 groundwater resources, where bagging models had a higher undertaken than the boosting models. Mosavi et al.^67^ presented four novel predictive methods, namely multivariate adaptive regression spline (MARS), flexible discriminate analyses (FDA), generalized linear model (GLM), and random forest (RF), for susceptibility mapping for erosion and flood. The results indicated that in modeling the flood susceptibility, the MARS, FDA, ensemble models, RF, and GLM, respectively, had the area under the curve (AUC) of 0.89, 0.92, 0.94, 0.93, and 0.91. Also, ensemble models, MARS, FDA, GLM, and RF, respectively, had an AUC of 0.97, 0.89, 0.92, 0.93, and 0.96 in determining erosion susceptibility. Mousavi et al.^68^ considered boosted regression trees (BRT) and RF as two ensemble ML models for the susceptibility mapping of groundwater hardness. The ensemble model results were compared to the multivariate discriminant analysis (MDA) model. The RF, BRT, and MDA models, respectively, showed improved performance based on the modeling findings. For a total set of 171 snow avalanches susceptibility mapping, Mosavi et al.^69^ proposed an ensemble ML model of random subspace (RS) based on a classifier functional tree. To evaluate the proposed model's goodness-of-fit and estimation accuracy, four benchmark models—logistic model tree (LMT), logistic regression (LR), functional trees (FT), and alternating decision tree (ADT)—were utilized. The presented ensemble model (RSFT), according to the results, performed better than the other soft-computing benchmark systems. Kamran^70^ demonstrated the use of ensemble learning techniques to forecast the drilling rate index (DRI) of rocks, such as the decision tree (DT), RF, and adaptive boosting (AdaBoost) with 57 data points, where Sievers' J-miniature drill value (Sj), Brazilian tensile strength, Uniaxial compressive strength (UCS), and brittleness test (S20) are some of the mechanical factors of rocks that are utilized as inputs to train the model. Among the ensemble learning approaches, the RF method has the best predictive performance for both the training (RMSE: 0.85 and MAE: 0.21) and testing (RMSE: 2.64 and MAE: 1.93) datasets. Kamran^71^ revealed a total of 62 datasets to estimate back-break (BB) using a state-of-the-art catboost-based t-distributed stochastic neighbor embedding (t-SNE) method. In the training and testing datasets, the BB estimation model based on t-SNE + Catboost has an RMSE = 0.260, MAE = 0.207, and RMSE = 0.282, MAE = 0.224, respectively. Shahani et al.^72^ developed four gradient boosting ML approaches including, extreme gradient boosting (XGBoost), Catboost, gradient boosted regression (GBR), and light gradient boosting machine (LightGBM) for estimating UCS of soft sedimentary rocks using a 106-point dataset. According to the findings, the XGBoost approach with statistical parameters such as RMSE: 0.00079 and MAE: 0.00062 in the training step and RMSE: 0.00069 and MAE: 0.00054 in the testing phase was affirmed to be the most precise approach among the four suggested approaches. Ullah et al.^73^ suggested three methods, including K-means clustering, XGBoost, and t-SNE to forecast the short-term rock-burst risk utilizing 93 rock-burst patterns with six authoritative characteristics from micro-seismic monitoring events. The outcomes of the suggested models provide an excellent benchmark for accurate prediction of future short-term rock-burst levels. Shahani et al.^74^ indicated the machine learning technology to estimate drilling rate index (DRI) of rocks, namely; the random forest algorithm (RFA), long short-term memory (LSTM), and simple recurrent neural network (RNN), where the LSTM method showed the best estimation of DRI with the lowest RMSE and highest R^2^ in the training (0.13416, 0.999) and testing (0.19479, 0.998) step, respectively.

Most of intelligent models are black box which means the mathematical relationship is not clear and explicit. These models mostly need special software such as Matlab or Python for their applications. On the other hand, white box models are those ones which generate explicit mathematical formula and can be easily applied by a simple calculator and they do not need any special software for their applications^75–77^. In the present research, two white box methods including genetic programming (GP) and group method of data handling (GMDH) have been used, which are explained in detail.

**Figure 1:**
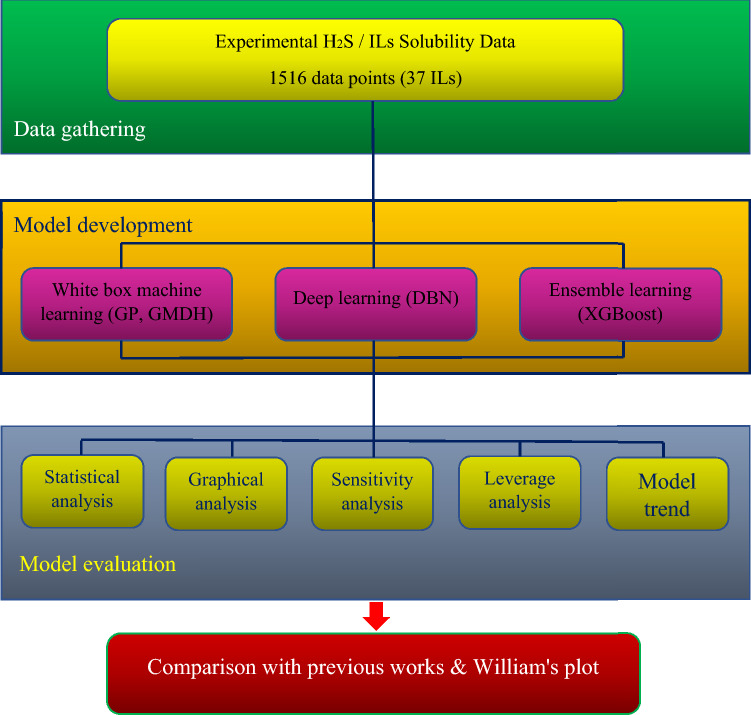
Flowchart of this study.

The main objective of this work is to establish exact models for computing H_2_S solubility in ILs using wider datasets and powerful techniques. In order to construct genetic programming (GP), group method of data handling (GMDH), deep belief network (DBN), and extreme gradient boosting (XGBoost), a large collection of 1516 experimental data from 37 ILs is employed. Seven thermodynamic features of ILs are chosen as inputs, including T, P, T_c_, P_c_, ω, M_w_, and boiling temperature (T_b_). Considering that this research aims to compare the proficiency of intelligent methods with equations of state, therefore, the selected parameters have been tried to have the same agreement with the required features of the EOSs so that we can have an accurate comparison among different methods. The sensitivity assessment is employed to figure out the relative effect of inputs on the H_2_S/ILs solubility. Finally, the validity of the models is evaluated utilizing William’s plot. Figure 1 shows the steps of this study. One of the circumscriptions that can exist in this research, like all studies based on artificial intelligence techniques, is their data-driven nature, which makes predictions reliable only in the range of experimental data, although the model may be used for new data. Therefore, the bigger the database, the more comprehensive and practical the model can be.

## Data collection and preparation

**Figure 2:**
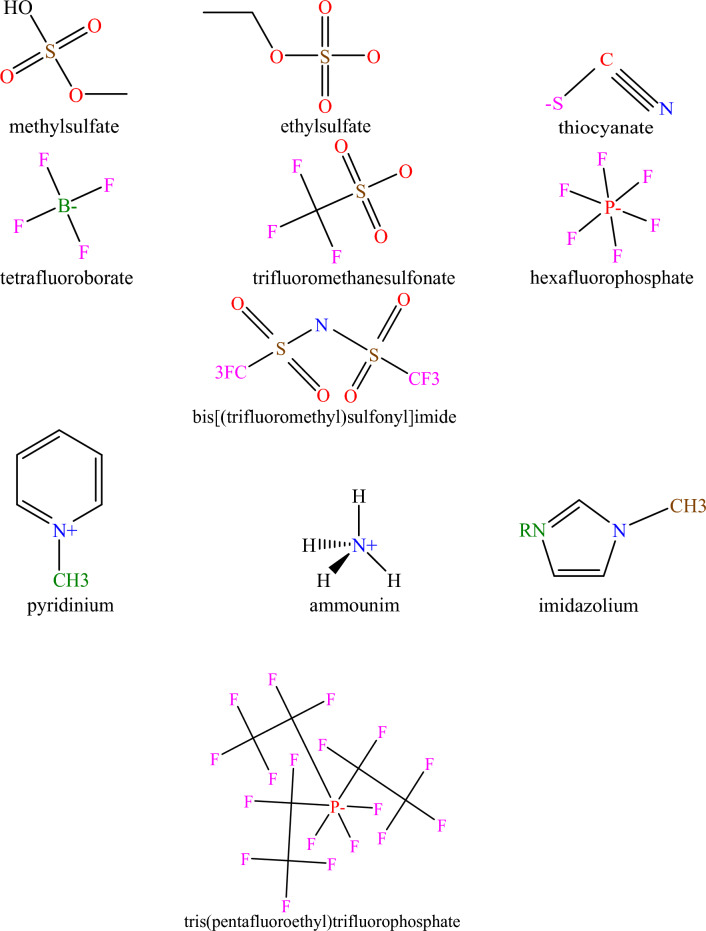
Chemical structures of different cations and anions of ILs^53^.

An extensive databank is essential for developing a predictive system with a high degree of trustiness and comprehensiveness. To create models, 1516 experimental data for H_2_S solubility of 37 ILs were acquired from the literature in the broad range of temperature range between 293.15 and 403.15 K and pressure between 0.001 and 96.3 bar. It is worth noting that H_2_S solubility in different ILs is from 0.0061 to 0.8900 mol fraction^3–7,14,15,26,34,53,57,62,63,78–90^. The provided data was apportioned into two categories before the model was developed. The training and test subsets comprise 80% and 20% of the whole data points, respectively. The test data was used to see how the generated system predict the output for new input parameters, while the training data was used to construct the original system. Features including T, P, T_c_, P_c_, ω, T_b_, and M_w_ were used as inputs, while H_2_S solubility was expressed as a mole fraction as an output. Table 1 highlights key details about the ILs we utilized to create our systems. Table 2 also shows the statistical evaluation information, such as the maximum, mode, minimum, and mean of the seven inputs, and H_2_S solubility employed in the current research. The chemical structures of the diverse anions and cations used in the current work are shown in Fig. 2.

**Table 1:** Characteristics of the experimental H_2_S solubility data used in the established models.

ILs	No. of data points	Temperature (K)	Pressure (bar)	H_2_S solubility (mole fraction)
[C_4_py] [BF_4_]	24	303.15–333.15	1.03–7.02	0.0350–0.3670
[C_4_py] [NTf_2_]	25	298.15–333.15	1.04–14	0.0670–0.8900
[C_6_MIM] [Ac]	66	293.15–333.15	0.003–3.309	0.0899–0.6093
[C_6_MIM] [BF_4_]	33	303.15–343.15	1.11–11	0.0600–0.4990
[C_6_MIM] [NTf_2_]	87	303.15–353.15	0.685–20.168	0.0290–0.7012
[C_6_MIM] [PF_6_]	34	303.15–343.15	1.38–10.9	0.0500–0.441
[C_8_MIM] [NTf_2_]	47	303.15–353.15	0.935–19.119	0.0630–0.7355
[C_8_MIM] [PF_6_]	48	303.15–353.15	0.845–19.584	0.0463–0.6972
[DMEAH] [Ac]	53	303.2–333.2	0.031–1.111	0.0104–0.2085
[DMEAH] [HCO_2_]	41	303.2–333.2	0.058–1.153	0.0065–0.1189
[MEDAH] [Ac]	35	303.2–333.2	0.097–1.396	0.0095–0.1618
[MEDAH] [HCO_2_]	33	303.2–333.2	0.079–1.242	0.0061–0.0807
[C_4_Py] [SCN]	21	303.15–333.15	1.04–6.22	0.0730–0.3540
[C_4_Py] [NO_3_]	23	303.15–333.15	1.3–6.8	0.0700–0.3480
[C_2_MIM] [NTf_2_]	43	298.15–353.15	1.077–16.86	0.0490–0.8100
[C_2_MIM] [PF_6_]	40	333.15–363.15	1.449–19.33	0.0320–0.3590
[C_2_MIM] [TfO]	36	303.15–353.15	0.643–24.553	0.0290–0.5672
[C_2_MIM] [TPTP]	79	303.15–353.15	0.582–19.415	0.0219–0.5926
[C_2_OHMIM] [BF_4_]	51	303.15–353.15	1.21–10.66	0.0200–0.2470
[C_2_OHMIM] [NTf_2_]	41	303.15–353.15	1.562–18.32	0.0575–0.5723
[C_2_OHMIM] [PF_6_]	47	303.15–353.15	1.336–16.85	0.0346–0.4627
[C_2_OHMIM] [TfO]	41	303.15–353.15	1.059–18.39	0.0356–0.5482
[C_4_MIM] [Ac]	69	293.15–333.15	0.001–3.415	0.0640–0.5789
[C_4_MIM] [BF_4_]	43	298.15–343.15	0.608–14	0.0300–0.79
[BDMIM] [NTf_2_]	1	298.15	14	0.79
[C_2_MIM] [Ac]	64	293.15–313.15	0.009–3.248	0.0916–0.5103
[C_2_MIM] [BF_4_]	59	298.15–353.15	0.508–18.483	0.0252–0.6057
[C_2_MIM] [CH_3_CH_2_CO_2_]	62	293.15–333.15	0.011–3.239	0.1205–0.5896
[C_2_MIM] [ESO_4_]	36	303.15–353.15	1.137–12.704	0.0120–0.1180
[C_2_MIM] [L-lactate]	57	293.15–333.15	0.044–3.216	0.0758–0.4897
[C_4_MIM] [Br]	1	298.15	1	0.0313
[C_4_MIM] [Cl]	2	298.15–358.15	14	0.86–0.87
[C_4_MIM] [MSO_4_]	8	298.1	0.108–7.509	0.0220–0.5210
[C_4_MIM] [NTf_2_]	45	298.15–343.15	0.944–14	0.0510–0.7700
[C_4_MIM] [PF_6_]	81	298.15–403.15	0.69–96.3	0.0160–0.8750
[C_4_MIM] [TfO]	39	298.15–343.15	1.693–16.32	0.0744–0.7800
[C_4_M'py] [NTf_2_]	1	298.15	14	0.85

**Table 2:** Statistical description of the prepared databank in the current study.

	Statistical parameters				
	Mean	Mode	Minimum	Maximum	Type
T (K)	321.97	313.15	293.15	403.15	Input
P (bar)	5.40	14.00	0.001	96.30	Input
Tb (K)	679.87	908.20	449.46	1084.84	Input
Tc (K)	939.57	1292.78	596.23	1479.45	Input
Pc (bar)	27.85	23.89	14.05	56.64	Input
w	0.69	0.39	0.22	1.46	Input
M (g/mole)	275.82	447.43	121	475.48	Input
H_2_S solubility (mole fraction)	0.25	0.07	0.01	0.89	Output

## Modeling

### Genetic programming (GP)

**Figure 3:**
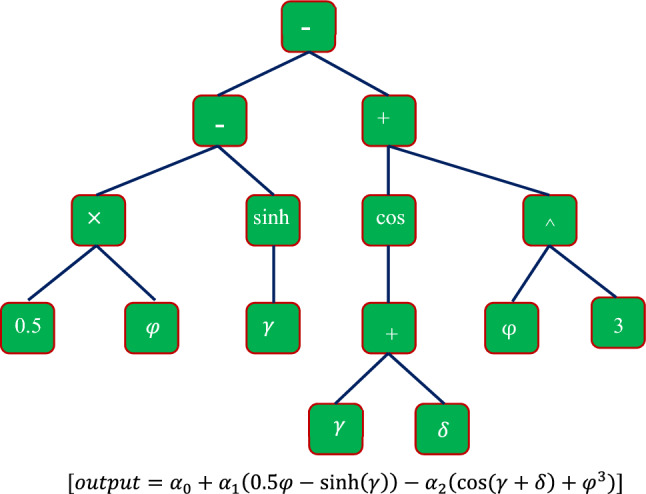
Schematic structure of a typical GP model.

In many engineering fields, Koza^91,92^ presented a novel evolutionary-based technique known as genetic programming (GP) for addressing issues automatically by introducing an expression characterizing the examined phenomena. Symbolic optimization will be used to identify possible answers utilizing the well-known design of tree illustration^91,92^. Terminals and functions are used in this sort of illustration, with functions being mathematical algebraic operators (e.g., +, −, log, exp) and terminals being solution factors and input parameters. The potential solution, which incorporates functions and terminals, may therefore be shown using an expression tree^91,92^. Several genetic operators, such as mutation, crossover, and selection, are used to increase the population of potential solutions regularly. This iterative procedure ends when the system's number of iterations is met or an acceptable solution is found. As a result, a fitness function is created to verify the solution quality, particularly using the mean square error (MSE)^93^, which must be reduced. The initial stage in computing a fitness function is to train the model, after which it may be used for forecasting^93^. Figure 3 depicts the schematic of the GP model.

### Group method of data handling (GMDH)

Ivakhnenko^94^ initially proposed the GMDH technique, which consists of mathematical methodologies and a black box nonlinear system characterization idea. He added a polynomial transfer function to the neuron, making it a more complicated unit. An automated technique for design procedure and weight modification was devised, and the links between layers of neurons were reduced. This approach may be thought of as a form of controlled Artificial Neural Network (ANN) that use natural selection to govern the system's size, intricacy, and precision. Simulation of complicated networks, function estimation, nonlinear regression, and pattern identification are the key applications of GMDH. The original model is created using the Volterra–Kolmogorov–Gabor (VKG) polynomial function as follows^94^:

1 $$y={a}_{0}+\sum_{i=1}^{m}{a}_{i}{x}_{i}+\sum_{i=1}^{m}\sum_{j=1}^{m}{a}_{ij}{x}_{i}{x}_{j}+\sum_{i=1}^{m}\sum_{j=1}^{m}\sum_{k=1}^{m}{a}_{ijk}{x}_{i}{x}_{j}{x}_{k}+\dots$$

In the above equation, $X=({x}_{1}, {x}_{2}, \dots )$, y, $A=({a}_{1}, {a}_{2}, \dots )$, and ${a}_{0}$ refer to the inputs vector, output vector, weights vector, and bias parameter, respectively.

The initial population of partial representations is created at the first stage of the system utilizing an algorithm that uses all conceivable combinations of two inputs. The sum of the two inputs $l=\left(\genfrac{}{}{0pt}{}{m}{2}\right)$ determines the total number of neurons produced on the first layer. At each stage, layers are generated depending on error indicators. At each stage of the layer production process, performance is assessed. The next layer starts with the largest number of neurons feasible, determines weights, and then freezes^95,96^. This differs from the back propagation approach, which allows all layers to engage in the training procedure simultaneously. In the sth repetition, the training of the GMDH system to create second-order polynomials is written in the following form^97^:

2 $$y=h\left(w, {X}^{\left(s\right)}\right) , s=1, \dots , m$$

Here *w*, *X*, and *m* signify the vector of factors in each neuron, the vector of system inputs, and the count of iterations of the system training step, respectively. The training and testing data are two subgroups of the sample dataset.

To recognize the count of neurons in each layer, a coefficient named "Selection pressure" should be specified as an appropriate threshold in this technique. After computing the parameters for all of the neurons, the ones that generate the worse results based on a previously set selection criterion should be deleted from the layer. RMSE is used as the threshold. The selection pressure can range from 0 to 1. All of the neurons had a similar configuration, with the best neurons being identified and progressing to the next step based on an external criterion. The precision of the obtained results may be impacted by the application of a critical threshold^97^.

3 $$\left\{\begin{array}{c}{e}_{c}=a{e}_{min}+(1-a){e}_{max}\\ e\le {e}_{c} \,\,(selection\,\, threshold)\end{array}\right.$$

**Figure 4:**
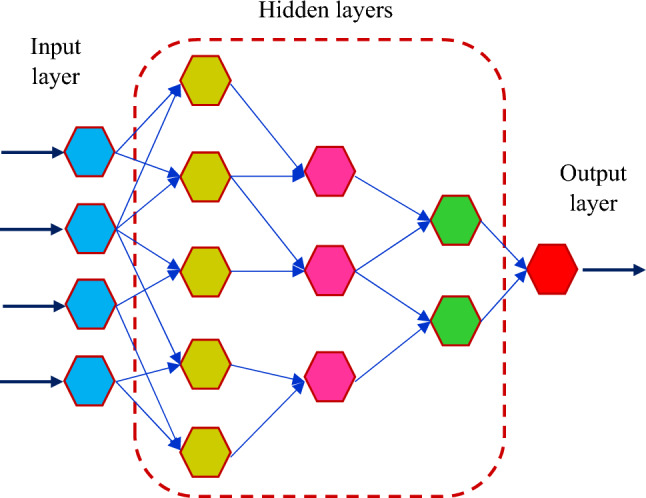
Schematic illustration of the GMDH model.

It is worth noting that just one neuron is chosen in the final layer. Figure 4 demonstrates the schematic structure of the GMDH model.

### Deep belief network (DBN)

**Figure 5:**
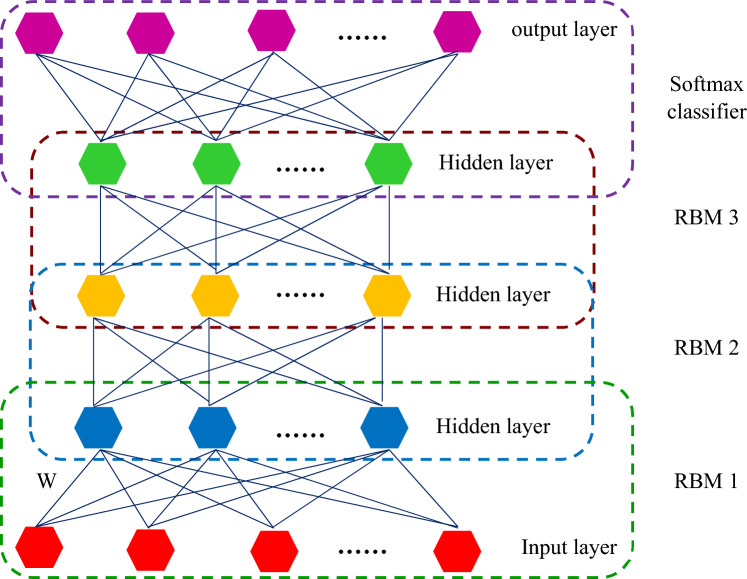
Schematic structure of the DBN model.

A DBN is a combination of RBMs that can be trained by moving the RBMs from the bottom to the top layer^98^. The RBM in the bottom layer trains the initial inputs and takes measurements; the extracted measurements, on the other side, will be the feed of another RBM in the top layer. By continuing this process, further layers of RBM can be produced^99^. Figure 5 depicts a common DBN system configuration. A DBN system's training procedure may be broken down into two parts^100^:

First stage: Uncontrolled train each RBM layer individually to ensure that feature information is preserved as much as feasible when feature vectors map to various feature regions.

Second stage: Adding a back propagation (BP) system to the top of the RBM's final layer, which takes the RBM's final layer's output feature vectors, and controlled training the BP system.

Since each layer of the RBM can only ensure that the weights of each layer well select to the input vectors of each layer for individual training, and the vector representation of the entire DBN system does not accomplish the desirable, the entire DBN system must be fine-tuned top-down by the BP system that is established on top. The procedure of fine-tuning the DBN may also be compared to the initialization of weights in a deep BP neural network. When the network trains the BP system, this strategy aids the training process prevent flaws like local optimum and considerable time spent in multi-level training. Initial training in deep learning is the first phase, and fine-tuning is the next stage in the aforementioned training model. Because the RBM can be quickly taught by evaluating divergence, the DBN reduces the complex deep learning neural network training phase by combining the training of several RBMs, making it more efficient than other deep learning neural network systems. A significant number of studies indicate that DBN can overcome the challenges that classic BP algorithms require a significant number of tag samples and have a sluggish convergence time while training multilayer neural networks^101^.

#### Restricted Boltzmann machines (RBM)

**Figure 6:**
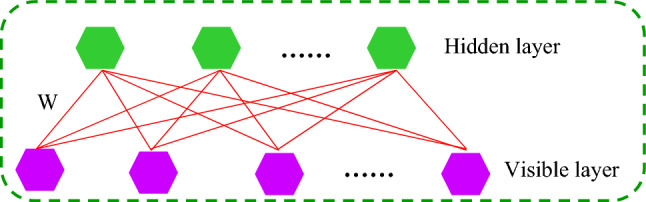
Structure of the RBM.

Figure 6 depicts the structural layout of the RBM. When the status of the input layer is specified, the activation criterion of each concealed layer is independent; on the other hand, when the status of the concealed layer is provided, the activation criterion of each transparent layer is independent^101,102^. RBM's system energy may be calculated as^103^:

4 $$E\left(v,h|\theta \right)=-\sum_{i=1}^{n}{a}_{i}{v}_{i}-\sum_{j=1}^{m}{b}_{j}{h}_{j}-\sum_{i=1}^{n}\sum_{j=1}^{m}{v}_{i}{w}_{ij}{h}_{j}$$

The activation possibility of the j^th^ concealed unit, according to the formula is^103^:

5 $$P\left({h}_{j}=1|v,\theta \right)=sigmoid({b}_{j}+\sum_{i}{v}_{i}{w}_{ij}$$

Similar manner, when the concealed unit is supplied, the ith transparent unit's activation possibility is^103^:

6 $$P\left({v}_{i}=1|h,\theta \right)=sigmoid({a}_{i}+\sum_{j}{h}_{j}{w}_{ij}$$

When training data are provided, an RBM's variables are adjusted to suit the provided training datasets. It may be expressed as optimizing the logarithm probability function using mathematical terms as follows^101^:

7 $$L\left(\theta \right)=log\prod_{t=1}^{T}P\left({v}^{(t)}|\theta \right)=\sum_{t=1}^{T}logP\left({v}^{(t)}|\theta \right)$$

### Extreme gradient boosting (XGBoost)

**Figure 7:**
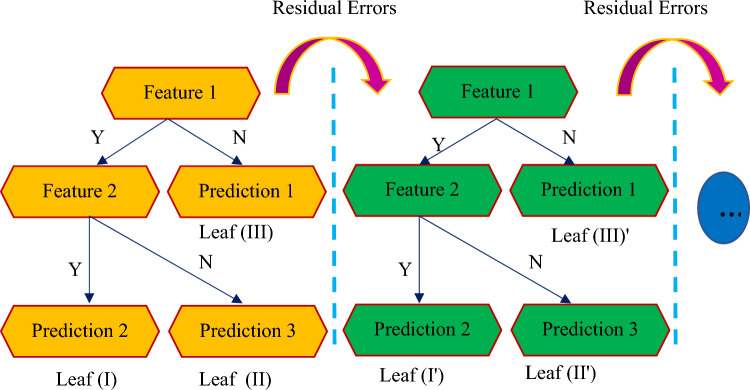
Schematic designation of the XGB model.

A tree-based entity approach's primary idea is to utilize a set of classification and regression trees (CARTs) to accommodate training sets utilizing a regularized objective function reduction. The XGBoost is a tree-based algorithm, which is component of the GBoost decision tree architecture. To better illustrate the CART establishment, each CART is consisting of root, internal, and leaf nodes, as demonstrated in Fig. 7. The first, which shows the complete data set, is separated into internal nodes, while the leaf nodes indicate the final categories, based on binary decision procedure^104^. A collection of n tresses is required to train and forecast the output y for a particular dataset, where m and n indicate dimension characteristics and instances, respectively^104^.

8 $$\begin{aligned} \hat{y}_{i} & = \sum\limits_{{k = 1}}^{N} {f_{k} } \left( {X_{i} } \right),\,\,f_{k} \in f \\ with\;f & = \left\{ {f\left( X \right) = \omega _{{q(x)}} } \right\},(q:R^{m} \to T,\omega \in R^{T} ) \\ \end{aligned}$$

In the above equation, the decision rule q(x) delineates the sample X to the binary leaf index. *f* denotes the area of regression trees, *f*_*k*_ the k^th^ independent tree, *T* the count of leaves on the tree, and *w* the weight of the leaf in Eqs. (1) and (2).

The reduction of the regularized objective function L: is employed to specified the collection of trees:

9 $$\begin{aligned} L = & \sum\limits_{i}^{n} l \left( {\hat{y}_{i} ,y_{i} } \right) + \sum\limits_{k}^{N} \Omega (f_{k} ) \\ with\;\Omega \left( f \right) = & \gamma T + \frac{1}{2}\lambda \parallel \omega \parallel ^{2} \\ \end{aligned}$$

Here $\Omega$ is the regularization parameter that aims to minimize overfitting by restricting system intricacy; *l* refers to a vicissitudinous convex loss function; $\gamma$ represents the smallest loss minimization required to cleave a new leaf; and $\lambda$ exhibits the regulation factor.

The objective function for each particular leaf is reduced in the gradient boosting strategy, and further leaves will be created repeatedly^105^.

10 $${L}^{(t)}=\sum_{i=1}^{n}\left\{l\left({y}_{i},{\widehat{y}}_{i}^{\left(t-1\right)}\right)+{f}_{t}({X}_{i})\right\}+\Omega ({f}_{t})$$

The tth repetition of the given training process is denoted by t. The XGBoost technique greedily increases the area of regression trees to significantly amend the ensemble system, which is represented to as a "greedy algorithm". Consequently, the result is modified repeatedly by minimizing the objective function:

11 $${\widehat{y}}_{i}^{(t)}={\widehat{y}}_{i}^{(t-1)}+{f}_{t}({X}_{i})$$

The XGBoost employs a shrinkage method that freshly affixed weights are adjusted by a learning component rate after each step of boosting. This reduces the danger of overfitting by limiting the influence of new further trees on each present particular tree^106^.

## Results and discussion

### Computational procedure

**Figure 8:**
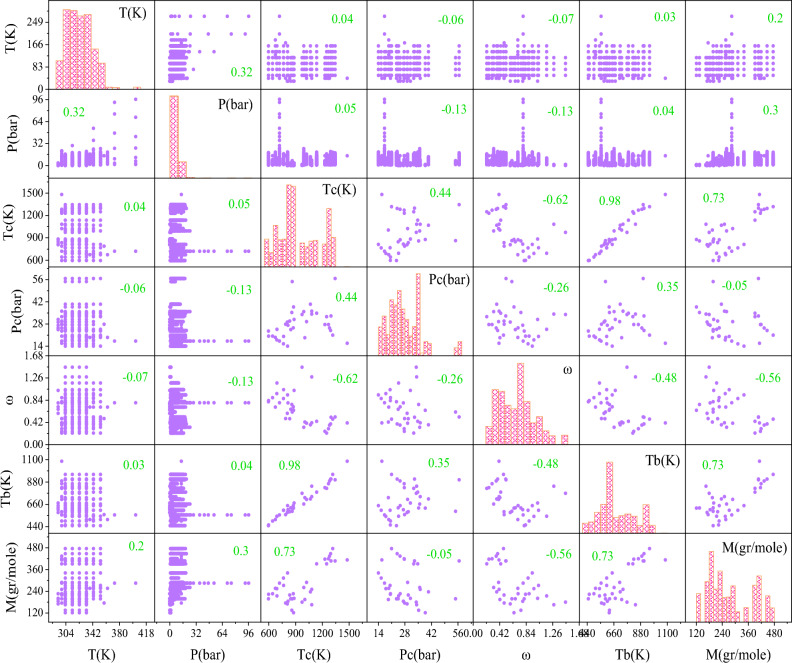
Pairwise correlation between input variables in this study.

To properly estimate the H_2_S/ILs solubility throughout a high diversity of thermodynamic conditions, 1516 data points of 37 ILs were gathered in this work. In this regard, four smart strategies were developed after selecting the inputs: T, P, T_c_, P_c_, ω, T_b_, and M_w_. Figure 8 illustrates the pairwise correlation plot of the inputs in this work. The categories for training and testing were created at random from the collected data. To develop the method and find the best model configuration, 80% of the data sets (1212 data points) were chosen at random as the training set. The train set was utilized to study the physical rules that existed in the systems. To assess the suggested models' accuracy and validity, the remaining 20% of the data sets that contained 314 data points were used. As previously stated, the suggested GMDH strategy is the simplest established method for estimating H_2_S solubility in ILs. This approach is expressed as simple mathematical formulas, such as:

$${H}_{2}S= -0.235902 + N9*2.37415 + N9*{N}_{1}*2.2592 - N9^2*5.60348 + {N}_{1}*0.496663 - {N}_{1}^{2}*0.159913$$

$${N}_{1} = -0.000632762 + {N}_{2}*0.537512 + {N}_{2}*{N}_{3}*1.98167 - {N}_{2}^{2}*0.875714 + {N}_{3}*0.419586 - {N}_{3}^{2}*0.935429$$

$${N}_{2} = -0.0909929 + {N}_{5}*0.559841 + {N}_{5}*{N}_{6}*1.0734 - {N}_{5}^{2}*0.342818 + {N}_{6}*0.914335 - {N}_{6}^{2}*1.05554$$

$${N}_{3} = -0.120569 + {N}_{8}*2.37521 + {N}_{8}*{N}_{4}*5.91963 - {N}_{8}^{2}*8.40394 - {N}_{4}^{2}*0.691399$$

$${N}_{4} = 1.43537 + {N}_{7}*0.867249 + {N}_{7}*{N}_{10}*0.988056 - {N}_{10}*13.1909 + {N}_{10}^{2}*28.1892$$

$${N}_{5} = -0.00923721 + {N}_{7}*1.05368 - {N}_{9}*1.5813 + {N}_{9}^{2}*5.65419$$

$${N}_{6} = 2.21984 + P *0.0233137 + P *{N}_{10}*0.0202401 - {P}^{2} *0.000236521 - {N}_{10}*19.0289 + {N}_{10}^{2}*40.9247$$

$${N}_{7} = 5.47619 + P *0.0809688 - P *T*0.000188643 - T*0.0293085 + {T}^{2}*3.95878e-05$$

$${N}_{8} = 6.03779 - T*0.0332957 + {T}^{2}*4.64602e-05 + w*0.500076 - {w}^{2}*0.425234$$

$${N}_{9} = 0.670851 - Pc *0.0183864 + Pc *w*0.0279851 - w*0.254225 - {w}^{2}*0.422828$$

$${N}_{10} = -0.0472412 + Tc *0.00187594 + Tc *M*7.44519e-06 - T{c}^{2}*2.12625e-06 - M*0.00356524 - {\left(M\right)}^{2}*6.28592e-06$$

In the aforementioned relationships, T, T_b_, and T_c_ are in (K), P and P_c_ are in (bar), ω is a unit-less variable, molecular weight is in (g/mole), and H_2_S solubility is in (mole fraction). As mentioned earlier, the developed GP smart model for computing H_2_S solubility in ILs is defined by the following correlations:

$${H}_{2}S=\left({c}_{0}T+{c}_{1}\right)exp({c}_{2}w))-{c}_{3} P(\left({c}_{4}-{c}_{5}\right)-{c}_{6})-(\left({c}_{7}Tc+\left(exp(exp \left({c}_{8}w\right) +{c}_{9}\right))\right)-\left(\frac{exp \left({c}_{10}w\right) }{\left(\left(ln(ln \left({c}_{11}{T}_{b}\right) +{c}_{12}\right))-\mathrm{exp}(exp \left({c}_{13}w\right)) \right)}+exp(exp \left({c}_{14}{P}_{c}\right)) \right){c}_{15}M)){c}_{16} +{c}_{17})$$

where

$${c}_{0}=1.0281;{c}_{1}=-11.658;{c}_{2}=2.1546;{c}_{3}=-0.23402;{c}_{4}=16.007;{c}_{5}=12.697;{c}_{6}=-11.658;{c}_{7}=1.8497;{c}_{8}=2.1546 ;{c}_{9}=5.0879;{c}_{10}=2.1546;{c}_{11}=11.06;{c}_{12}=19.674;{c}_{13}=2.1546;{c}_{14}=-0.21254;{c}_{!5}=-0.38433;{c}_{16}=0.0024311;{c}_{17}=1.6133$$

### Statistical assessment of the developed methods

Statistical criteria were employed to assess the precision and vigorous of the suggested techniques in forecasting H_2_S/ILs solubility. The statistical variables used in the analysis were average absolute percent relative error (AAPRE%), root mean square error (RMSE), coefficient of determination (R^2^), standard deviation (SD), average percent relative error (APRE%), and a-20 index. The mathematical expression of the aforementioned criteria is as below:

1. Average absolute percent relative error:
  12 $$AAPRE=\frac{1}{n}\sum_{i=1}^{n}\left|\{({S}_{i exp.}-{S}_{i pred.})/ {S}_{i exp.}\}\times 100\right|$$
2. Root mean square error:
  13 $$RMSE= \sqrt{\frac{1}{n}{\sum }_{i=1}^{n}{\left({S}_{i exp.}-{S}_{i pred.}\right)}^{2}}$$
3. Correlation coefficient:
  14 $${R}^{2}=1-\frac{{\sum }_{i=1}^{n}{\left({S}_{i exp.}-{S}_{i pred.}\right)}^{2}}{{\sum }_{i=1}^{n}{\left({S}_{i exp.}-{\overline{S} }_{i exp.}\right)}^{2}}$$
4. Standard deviation:
  15 $$SD=\sqrt{\frac{1}{n-1}\sum_{i=1}^{n}{\left(\frac{{S}_{i exp.}-{S}_{i pred.}}{{S}_{i exp.}}\right)}^{2}}$$
5. Average percent relative error:
  16 $$APRE=\frac{1}{n}\sum_{i=1}^{n}[({S}_{i exp.}-{S}_{i pred.})/ {S}_{i exp.}]\times 100$$
6. a-20 Index:
  17 $$a-20\,\, Index=\frac{m20}{n}$$

where, the count of data points in the original data set is denoted by n. ${S}_{ipred.}$ and ${S}_{iexp.}$, respectively, state the estimated and measured values of ith data. ${\overline{S} }_{iexp}$ defined the mean value of experimental data. The parameter m20 refers to the samples with a value of rate exp./pred. value between 0.8 and 1.2.

Table 3 indicates the computed values for these variables for the testing, training, and the whole dataset of all paradigms. The findings show that XGBoost gives the highest accurate predictions of all the evaluated models. Indeed, the XGBoost paradigm has the highest precise prediction of H_2_S/ILs solubility, with AAPRE values of 1.14% for the all data-points, 1.30% of testing set, 1.09% for the training.

**Table 3:** Statistical results of the developed smart models in the present study.

Models	APRE (%)	AAPRE (%)	RMSE	SD	R^2^	a-20 Index
XGBoost						
Train	− 0.0526	1.0995	0.0023	0.0184	0.9998	1
Test	− 0.0285	1.3038	0.0027	0.0237	0.9998	1
Total	− 0.0478	1.1404	0.0024	0.0196	0.9998	1
DBN						
Train	− 4.9838	7.7231	0.0128	0.1567	0.9944	0.94
Test	− 6.1407	8.9410	0.0119	0.2105	0.9959	0.92
Total	− 5.2158	7.9673	0.0126	0.1688	0.9947	0.94
GP						
Train	2.1159	26.1676	0.0860	0.5056	0.7404	0.53
Test	6.9539	23.3151	0.0509	0.4252	0.9145	0.54
Total	3.0829	25.5975	0.0802	0.4906	0.7867	0.53
GMDH						
Train	6.3556	14.0833	0.0537	0.1979	0.8986	0.75
Test	20.4613	25.6994	0.0709	0.3445	0.8341	0.52
Total	9.3406	16.5551	0.0577	0.2410	0.8895	0.71

### Graphical analysis of the models

In addition to the presented error evaluation criteria, four graphical analyses were provided in this research to assess the effectiveness of the recommended paradigms. A succinct description of these charts is presented as follows:

1. Cumulative frequency: In this graph, the cumulative frequency is shown versus the absolute relative deviation. The percentage of various error intervals is presented.
2. Error bar: In this graph, the AAPRE of the suggested approaches is shown by bars.
3. Cross-plot: The estimated value is displayed against the experimental value. In such a plot, the tight collection of data points along the Y = X line suggests that the model is the most correct.
4. Taylor diagram: It demonstrates which of the numerous plausible representations of a system or phenomenon is the most realistic. The degree of connection between the estimated and real behavior is measured using three statistical criteria: RMSE, SD, and R^2^.

**Figure 9:**
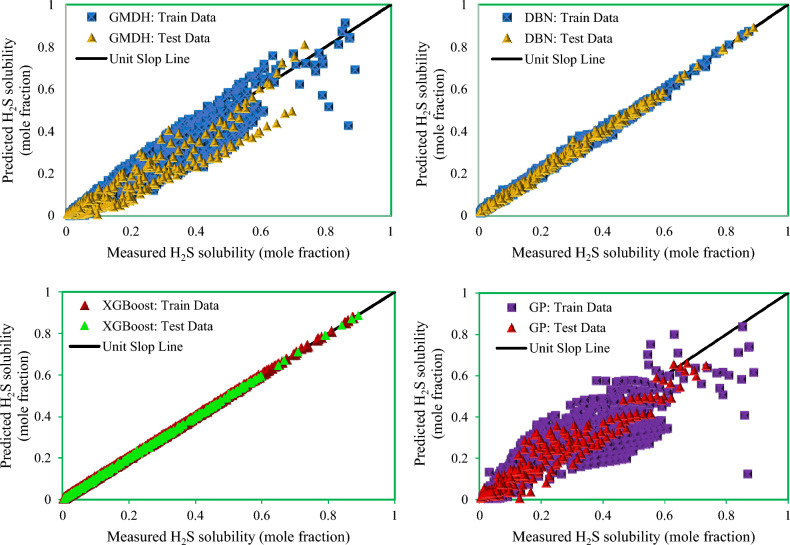
Cross plot of the proposed methods in this study for estimation of the H_2_S solubility in ILs.

**Figure 10:**
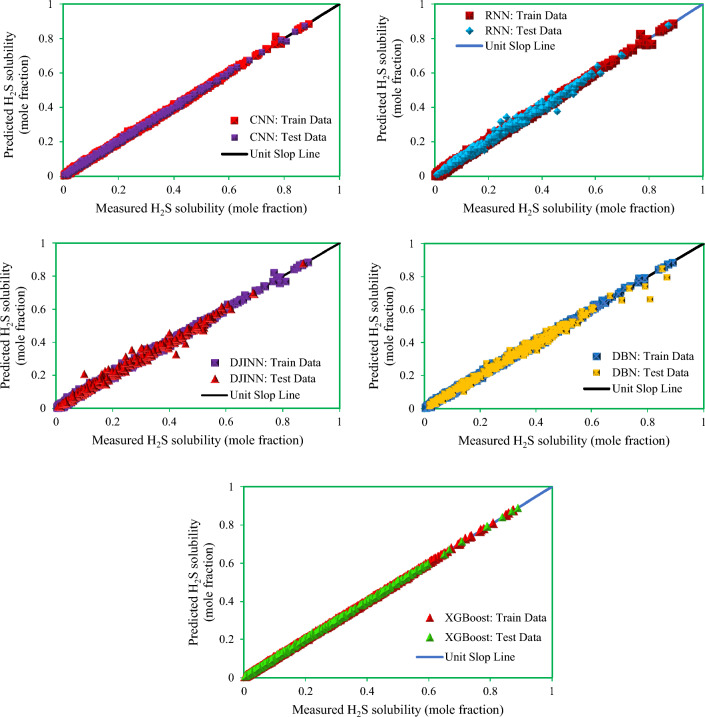
Cross plots of the proposed XGBoost model in this study and previous developed techniques for estimating the H_2_S solubility in ILs.

**Figure 11:**
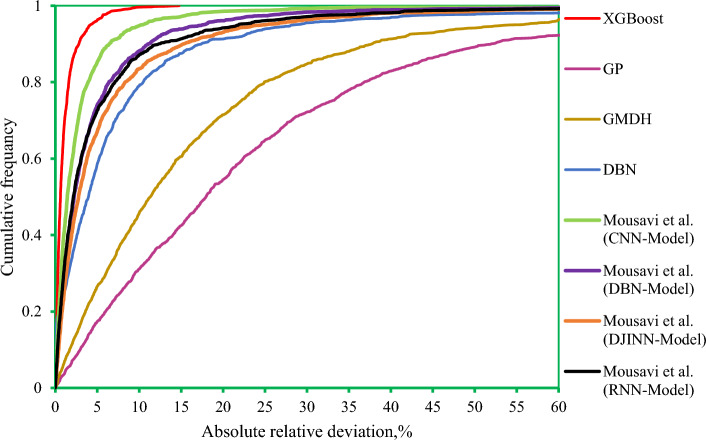
Cumulative frequency of absolute relative deviation in different models in this study and previous works.

**Figure 12:**
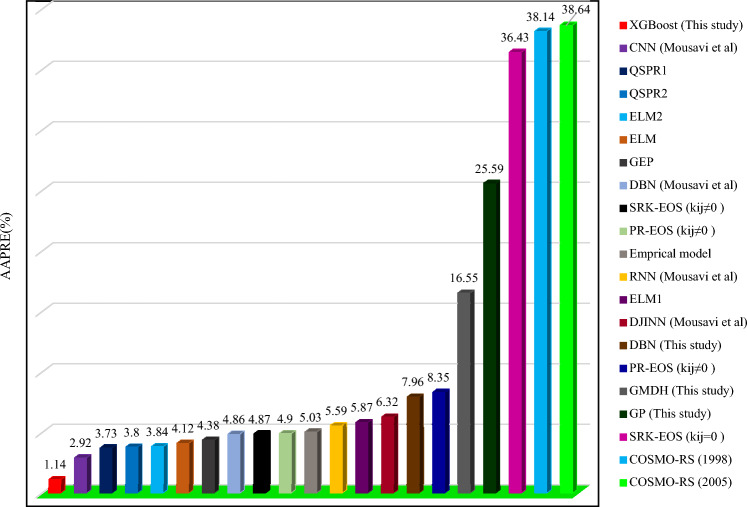
Comparison between AAPRE of different models for estimating the H_2_S solubility in different ILs.

**Figure 13:**
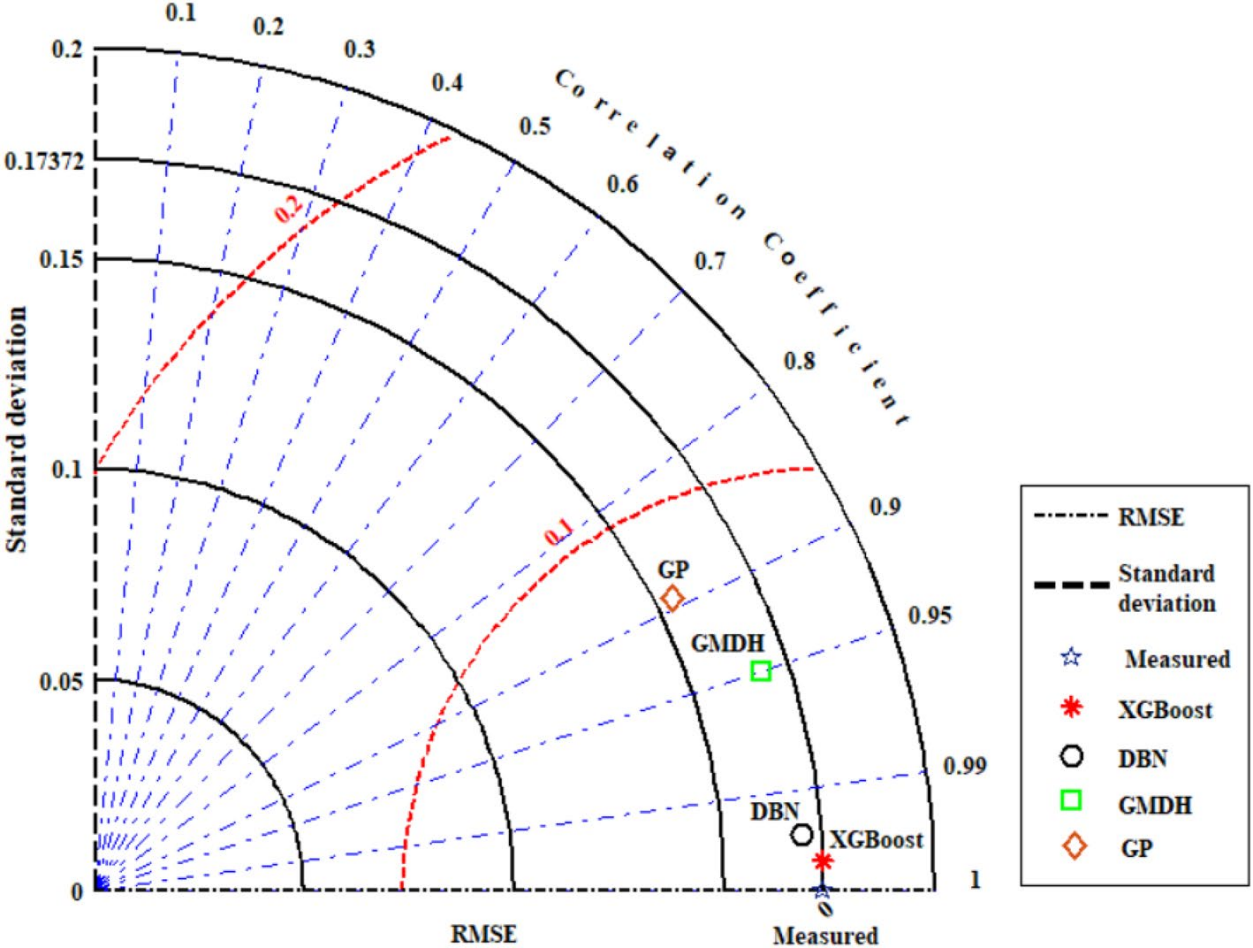
Taylor diagram for the proposed paradigms namely GP, GMDH, DBN, and XGBoost based on thermodynamic properties, temperature, and pressure.

Figure 9 shows cross plots of actual values against predicted values for the four techniques utilized in this study, namely DBN, GMDH, GP, and XGBoost. Around the unit slope line, there is a considerable dispersion of data points, both testing and training ones, indicating that three smart models, GP, DBN, and GMDH, have an improper match between predicted and experimental data points. As can be shown from this chart, a tight aggregation of entire dataset is placed around the Y = X line for both training and testing sets for the developed XGBoost model. Indeed, the XGBoost approach outperforms other models in terms of accuracy and performance, and it can estimate the hydrogen sulfide/ILs solubility with less variation from real data (the same results as reported in Table 3). Cross plots of an estimated H_2_S solubility in ILs versus experimental data are displayed in Fig. 10 to compare the forecasting ability of the XGBoost model as the best model against available methods in the Mousavi et al.^53^ study, namely, CNN (R^2^_train_: 0.9991 and R^2^_test_: 0.9989), DBN (R^2^_train_: 0.9983 and R^2^_test_: 0.9912), and DJINN (R^2^_train_: 0.9975 and R^2^_test_: 0.9868). The previously developed techniques' data points are scattered around the unit slope line in this figure, but the established XGBoost (R^2^_train_: 0.9998 and R^2^_test_: 0.9998) model's training and test set data points are tightly gathered around the unit slope line. This reveals the developed XGBoost model's high precision as well as the inability of previous approaches to calculate H_2_S solubility in ILs. In the following step, Fig. 11 shows a comparison of the present study results and introduced methods by Mousavi et al.^53^. As this figure demonstrates, about 96% of XGBoost, 86% of CNN^53^, 75% of DBN and RNN models^53^, 70% of DJINN^53^, 57% of DBN, 26% of GMDH, and17% of GP predictions have errors less than 5%. This implies that the XGBoost smart method is the most practical method for calculating H_2_S solubility in ILs among all previously investigated techniques. Figure 12 compares the AAPRE % of the literature models to the smart methodologies employed in this work to analyze the model's capabilities. Examples of available techniques are including ELM1 (1318 measured data for 28 ILs)^34^, COSMO-RS (722 (15 ILs) measured data- ADF 2005)^57^, GEP (465 measured data for 11 ILs)^58^, SRK-EOS (465 (11 ILs) measured data, kij ≠ 0)^58^, SRK-EOS (kij = 0, 465 (11 ILs) measured data)^58^, PR-EOS (kij ≠ 0, 465 (11 ILs) measured data)^58^, COSMO-RS (722 (15 ILs) measured data—ADF 1998)^57^, Empirical model (664 measured data for for 14 ILs)^89^, QSPR1 (1282 measured data for 27 ILs)^35^, PR-EOS (kij ≠ 0, 664 (14 ILs) measured data)^89^, ELM (1282 data points for 27 ILs)^62^, ELM2 (1318 measured data for 28 ILs)^34^, QSPR2 (27 ILs—1282 data points)^35^, and Mousavi et al.^53^ which developed four smart models namely CNN, DBN, RNN, and DJINN for estimation H_2_S with a large databanks (1516 data-point in 37 various ILs). In compared to previous techniques for computing H_2_S solubility in ILs based on thermodynamic parameters, the XGBoost (AAPRE = 1.14%) model is clearly the most accurate and adaptable. Furthermore, Fig. 13 introduced Taylor chart, which combines a variety of evaluation criteria for a more intelligible design. This graphic illustrates all of the intelligent models in terms of how well they account H_2_S solubility in ILs. The RMSE, SD, and R^2^ of systems, namely XGBoost, DBN, GP, and GMDH, are implemented to quantify the difference between the calculated and actual data. The centered RMSE is calculated using a distance from the total measure as the reference point. The consummate predictive system, which is indicated by a point with R^2^ equal to 1, is next criterion in the Taylor diagram. The RMSE and R^2^ values for the developed XGBoost smart method in this study were 0.0024 and 0.999, respectively. The statistical parameters for the smart methods in the current study are shown in Fig. 13 after the models have been put into the Taylor diagram. Based on how far away from the desire instance, which is labeled as "measured" in this chart, each paradigm's overall performance is evaluated. The Taylor diagram further emphasizes XGBoost's domination because it is the closest to actual measurements when evaluating performance globally.

### Sensitivity analysis

In the current survey, a sensitivity assessment was accomplished to detect the influence of each input variable (i.e., P, T, P_c_, T_c_, ω, M_w_, and T_b_) on the output prediction using the XGBoost as the best model developed. In this regard, the relevancy factor (r) was used to determine the degree of each parameter's effect on the H_2_S solubility in ILs as an output parameter^55,107^. The r value is shown below:

18 $$\mathrm{r}\left({\mathrm{inp}}_{\mathrm{i}},\mathrm{O}\right)= \frac{{\sum }_{\mathrm{j}=1}^{\mathrm{n}}\left({\mathrm{inp}}_{\mathrm{i},\mathrm{j}}-{\overline{\mathrm{inp}} }_{\mathrm{i}}\right)\left({\mathrm{O}}_{\mathrm{j}}-\overline{\mathrm{O} }\right)}{\sqrt{{\sum }_{\mathrm{j}=1}^{\mathrm{n}}{\left({\mathrm{inp}}_{\mathrm{i},\mathrm{j}}-{\overline{\mathrm{inp}} }_{\mathrm{i}}\right)}^{2}{\sum }_{\mathrm{j}=1}^{\mathrm{n}}{\left({\mathrm{O}}_{\mathrm{j}}-\overline{\mathrm{O} }\right)}^{2}}}$$

here $\overline{\mathrm{O} }$ and ${\mathrm{O}}_{\mathrm{j}}$ are the mean jth values of the forecasted parameters (O $=$ H_2_S solubility in ILs), respectively. While, ${\mathrm{inp}}_{\mathrm{i}}$ and ${\mathrm{inp}}_{\mathrm{i},\mathrm{j}}$ represent the mean of the ith input parameter and the jth value, respectively (${\mathrm{inp}}_{\mathrm{i}}$ is P, T, P_c_, T_c_, ω, M_w_, and T_b_).

The relevance factor (r), which ranges from [− 1, 1], depicts the impression of inputs on the model's output as follows:

1. If $\left(r>0\right)$, each of the input variables has an increasing effect on the predicted parameter. In other words, the output value increases when the desired parameter is increased. As a result, the larger the impact, the closer the relevance factor is to 1.
2. If $\left(r=0\right)$, the output data and the input parameters have no relationship or this is increasing a region and decreasing in another region.
3. If $\left(r<0\right)$, the input variable's effect on the output is decreasing. Enhancing the targeted variable decreases the output parameter's value. The larger the effect, the closer the relevance factor is to − 1.

**Figure 14:**
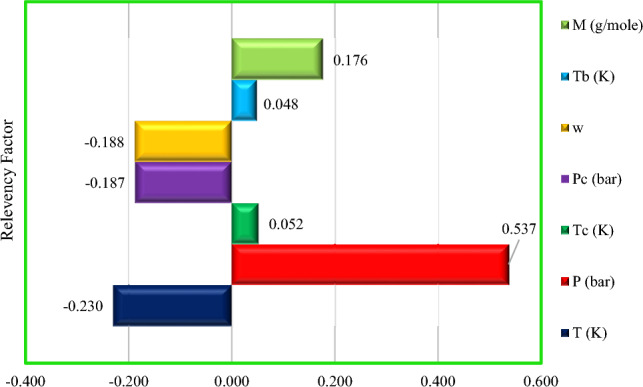
Relative impact of each input variable on the H_2_S solubility in ILs as output.

The relevancy factor value for the XGBoost model's inputs as the best paradigm is indicated in Fig. 14. According to this figure, each of input parameters including P, M_w_, T_b_, and T_c_ have direct effects on H_2_S solubility, whereas T, P_c_, and ω have contrary effects. This means that changing the value of each input will change the value of H_2_S solubility. Among the positive input parameters, the pressure with a relevancy factor of + 0.537 has the greatest impact. As a result, the solubility of H_2_S increased as pressure increased. This phenomenon is explained by Henry's law, which expresses that the gas solubility in liquids is proportional to the pressure of that gas above the solution's surface. Gas molecules are driven into the solution as pressure increases, therefore this leads to higher amount of gas molecules dissolve in the solution^58^. On the other hand, the temperature with a relevancy value of − 0.230 has the highest influence through the negative input variables. This implies that when the temperature is raised, the solubility of H_2_S decreased. Consequently, heating the solution creates thermal energy, which overcome the gas/solvent molecules attraction, decreasing the gas solubility^53^.

### Applicability domain of the XGBoost method

**Figure 15:**
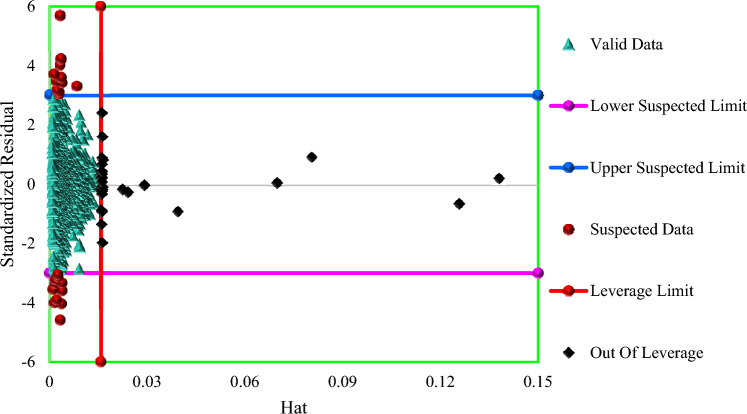
The William's plot of the whole dataset for XGBoost model to identify the applicability domain.

To evaluate the validity region of the suggested XGBoost system and to distinguish any data that is dubious, the Leverage approach was utilized^108–110^. The Leverage evaluation, that has been formed graphically through Williams' plot, is one of the most substantial methods of outlier distinction. This method includes computing residuals and the Hat matrix for the input data. The deviation of actual data and method predictions is represented by residual values (R), while the Hat matrix is determined as indicated here^111^:

19 $${\mathrm{H }=\mathrm{ X}({X}^{t}X)}^{-1} {X}^{t}$$

where *X* is an (a × b) matrix, where, a is the number of data points and b is the number of method variables, ${X}^{t}$ is the transpose of the matrix *X*. Furthermore, H* (the Leverage limit) is calculated as $\frac{3(a+1)}{b}$. In this study, the H* value for the XGBoost model was 0.015. The R values are then plotted against the hat variable to produce the Williams plot, which shows the suspicious data as well as the usable region of the model. The developed paradigm is regarded authentic and its forecasts are performed in the validity scope if majority of the data points were collected between the ranges of 0 ≤ *H* ≤ *H*^∗^, and − 3 ≤ *R* ≤ 3. Figure 15 shows the Williams plot of the XGBoost method. Virtually all data points in Fig. 15 appeared to be between 0 ≤ *H* ≤$0.015$ and − 3 ≤ *R* ≤ 3. Therefore, only 56 data points are over and above the aforementioned range, according to the outlier’s identification findings. Indeed, 24 data points of whole data set (1.58% of databank) are suspected data, and the rest of the data (32 data points, 2.11% of all data) are outside the allowed leverage (0.015 ≤ H). The findings in Fig. 15 show that the XGBoost model used to predict H_2_S in ILs is statistically valid, and the datasets used to create the model are of sufficient quality to be used.

### Qualitative investigation of H_2_S solubility in ILs

**Figure 16:**
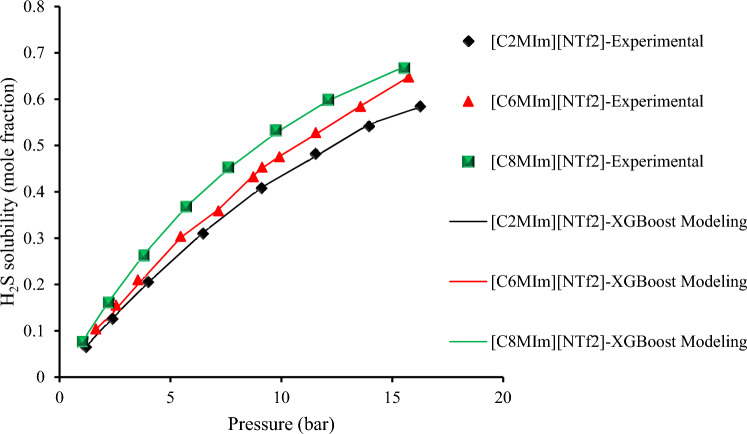
The influence of cation alkyl chain length on the H_2_S solubility with the same anion (at 313.15 K).

**Figure 17:**
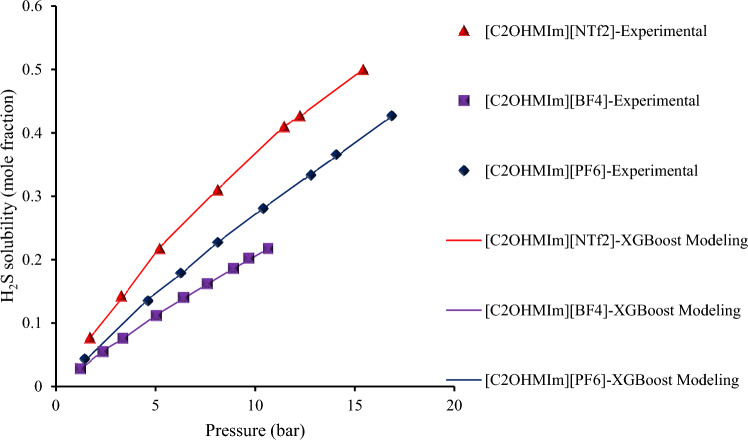
The impact of the fluorine content in anion on the H_2_S solubility in ILs with the same cation (at 313.15 K).

Hydrogen sulfide/ILs solubility increases with rising pressure and decreasing temperature, and a similar pattern is also evident for CO_2_ solubility in ionic liquids^112^. This gas dissolves due to its molecules interact with those of the liquid. Since heat is generated when these new attractive interactions create, dissolution is an exothermic process. Thermal energy is created when heat is applied to a solution, which prevails the gas/liquid molecules attractions, decreasing the gas solubility. In compared to the anion, the cation has a minor effect on H_2_S solubility. Figure 16 shows the influence of cation alkyl chain length on H_2_S solubility at 313.15 K with an identical anion. In comparison to ILs with shorter chains, those with longer alkyl chains may have greater H_2_S solubility ([C_8_MIm]^+^ > [C_6_MIm]^+^ > [C_2_MIm]^+^). This effect is explained by the verity that ILs with longer alkyl chains have larger free volumes, which allows them to raise van der Waals interactions while also admitting more H_2_S molecules^5,6,62^. Figure 17 shows the influence of fluorine content in anion on H_2_S solubility at 313.15 K for three ILs with various kinds of anions but the same cation. Three ILs are including: 1-(2-hydroxyethyl)-3-methyl-imidazolium bis(trifluoromethylsulfonyl)amide [C_2_OHMIM] [NTf_2_], 1-(2-hydroxyethyl)-3-methyl-imidazolium hexafluorophosphate [C_2_OHMIM] [PF_6_], and 1-(2-hydroxyethyl)-3-methyl-imidazolium trifluoromethanesulfonate [C_2_OHMIM] [TfO]^62,63^. As a result, increased H_2_S solubility is linked to higher fluorine content in anion ([NTf_2_] > [PF_6_] > [BF_4_])^5,6^. Finally, based on Figs. 16 and 17, it can be stated that the constructed XGBoost could predict this phenomenon as an accurate and highly reliable model.

### Pressure trend analysis of the developed XGBoost approach

**Figure 18:**
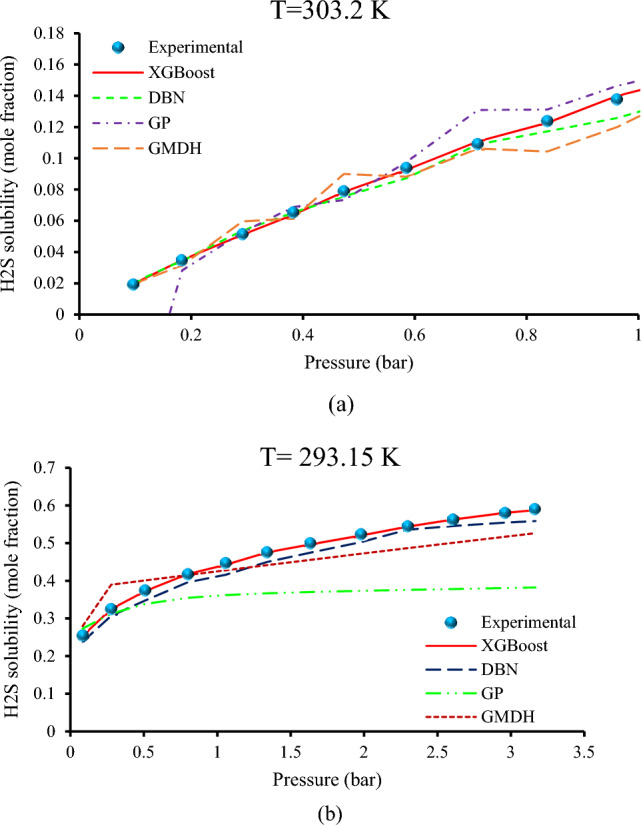
Trend investigation of the developed XGBoost model in predicting H_2_S solubility in various ILs with regard to (**a**) [MEDAH] [Ac], (**b**) [C_2_MIM] [CH_3_CH_2_CO_2_].

Different visual evaluations were performed as the last assessment step to examine the XGBoost model's capacity in the H_2_S solubility in various ILs. The validity of developed models, on the other hand, may be assessed by comparing the trend of measured variations to experimental and estimated data. Figure 18 illustrates the effect of pressure on H_2_S solubility for two different ILs, methyldiethanolammonium acetate [MEDAH] [Ac] and 1-ethyl-3-methylimidazolium propionate [C_2_MIM] [CH_3_CH_2_CO_2_], at temperatures of 303.2 K and 293.15 K, respectively. Figure 18 depicts that the suggested XGBoost model was able to correctly determine the physical trend of the behavior, as demonstrated in this diagram. The H_2_S becomes more soluble in ILs as the pressure increases. This phenomenon is explained by Henry's law^58^. Gas molecules are compelled into the solution as pressure rises, therefore this leads to a higher quantity of gas molecules incorporated in the solution. As a result, based on thermodynamic characteristics, temperature, and pressure, the proposed technique may produce exact and reliable predictions for the H_2_S solubility in various ILs. As previously stated, Fig. 14 depicts a sensitivity assessment of the inputs and output of the XGBoost. Pressure has a favorable influence on H_2_S solubility in various ILs, which is consistent with the findings shown in Fig. 18.

## Conclusions

In the current study, 1516 real data points were used to forecast the H_2_S solubility in ILs. A comprehensive databank was prepared from literature and includes variables such as T, P, T_c_, P_c_, ω, T_b_, and M_w_. In this work, four intelligent models—DBN, GMDH, GP, and XGBoost—were developed to compute the H_2_S solubility in ILs. The statistical indices (namely; AAPRE, APRE, RMSE, SD, and R^2^) and graphical analyses (such as; cross-plot, Tylor diagram, error chart, and cumulative frequency) findings of these recently implemented tools were applied to assess the achievements of the suggested methods. The following findings are found from this research:

1. The H_2_S solubility in ILs was estimated using four models in the current study, of which XGBoost was determined to be the most precise and valid.
2. The newly established XGBoost model for computing the H_2_S solubility in ILs systems is smart enough to make very accurate predictions about the H_2_S solubility values (total AAPRE: 1.14%, train AAPRE: 1.09%, and test AAPRE: 1.30%).
3. Furthermore, the XGBoost is more accurate and trustworthy when results are compared to those of other well-known earlier models.
4. The relevancy factor was applied in order to examine how each input parameter affected the H_2_S solubility. Pressure (based on Henry’s law) has a more favorable impact on model results than the other input variables for the XGBoost method. While the solubility of H_2_S is inversely related to temperature.
5. According to the leverage technique, the majority of the data are empirically valid, and only a small number of data points are found outside the XGBoost model's application area.
6. In terms of pressure, the XGBoost model's output trends make logical.
7. The developed XGBoost model is not only quick and inexpensive, but it also accurately predicts the solubility of H_2_S.

## Data Availability

All the data have been collected from literature. We cited all the references of the data in the manuscript. However, the data will be available from the corresponding author on reasonable request.
